# Exploring Higher Education Pathways for Coping With the Threat of COVID-19: Does Parental Academic Background Matter?

**DOI:** 10.3389/fpsyg.2021.768334

**Published:** 2022-01-07

**Authors:** Julius Möller, J. Lukas Thürmer, Maria Tulis, Stefan Reiss, Eva Jonas

**Affiliations:** Department of Psychology, University of Salzburg, Salzburg, Austria

**Keywords:** COVID-19, defensive strategies, first-generation students, system justification, social belonging

## Abstract

First-generation students (FGS) are more likely to feel misplaced and struggle at university than students with university-educated parents (continuous-generation students; CGS). We assumed that the shutdowns during the Coronavirus-pandemic would particularly threaten FGS due to obstructed coping mechanisms. Specifically, FGS may show lower identification with the academic setting and lower perceived fairness of the university system (system justification). We investigated whether FGS and CGS used different defenses to cope with the shutdown threat in a large sample of German-speaking students (*N* = 848). Using Structural Equation Modeling, we found that for all students, independent of academic parental background, high levels of system justification were associated with perceiving the learning situation as less threatening, better coping with failure, and less helplessness. However, in comparison to CGS, FGS showed small but significant reductions in system justification and relied more on concrete personal relationships with other students as well as their academic identity to cope with the threatening situation. We discuss implications for helping FGS succeed at university.

## Introduction

Educational pathways are filled with challenges and obstacles for every student, independent of their socio-demographic background. Attaining an academic degree means having to overcome a number of barriers and taking advantage of the right opportunities at the right time. However, for some students these hurdles can be harder to face than for others. One group of students who might face unique challenges in the academic context are first-generation students (FGS). Coming from families where no parent achieved an academic degree places them in a different situation than students whose parents attained post-secondary education (continuous-generation students, CGS).

This situation might have been particularly challenging with the COVID-19 pandemic posing a tremendous threat not only to society in general, but especially to education systems as institutions were ordered to close from spring 2020 (Aristovnik et al., 2020). Universities were forced to swiftly transition to only online teaching, providing a great challenge for universities, faculties, and especially students (Oliveira et al., 2021). Not only did the learning environment transfer from university grounds’ back into the students’ bedrooms, but social contact with other students was reduced to a bare minimum or vanished completely. Subsequently, the two key elements of student identification, contact with peers and the university environment (Barber et al., 2021), were no longer available during lock-down. This sudden and urgent change caused uncertainty for all involved (Jeong et al., 2021).

The loss of social interaction with other students might harm FGS more than CGS as they have fewer inherent connections to the academic system. Accordingly, the resulting social isolation of pandemic living conditions may have led FGS to struggle more with their studies than CGS during this time (Terenzini et al., 1996; Pascarella et al., 2004; Soria and Stebleton, 2012; Stebleton and Soria, 2013). The transition to online learning might therefore have had a disproportionate impact on FGS and their identification as a student, leading them to feel especially isolated and abandoned by the university to face their academic challenges alone. Thus, they potentially perceived the university system as more unfair and disadvantageous than CGS, resulting in blaming the university for the threatening situation. Our aim in the present study was to investigate whether FGS and CGS differ in how they coped with the threat the COVID-19 pandemic had on their educational progress. Therefore, we investigated if FGS and CGS differentiate in their use of strategies such as justifying the university system, relying on their academic identity, and capitalizing on their peers (i.e., social belonging).

### First-Generation Students

With the steady increase of student numbers in Austria in recent years, students’ backgrounds have become more diverse (Institut für Höhere Studien, 2021). In addition to ethnic or socioeconomic differences, the differentiation between FGS and CGS has gained interest. FGS can be defined as students at the tertiary level of education whose parents did not achieve a post-secondary degree (Spiegler and Bednarek, 2013). Extensive research has portrayed FGS as a vulnerable group, with deficits regarding their academic achievement compared to their peers with parental academic backgrounds (Spiegler and Bednarek, 2013). FGS are less likely to graduate from university than CGS (Soria and Stebleton, 2012; Cataldi et al., 2018). Twenty-seven percent of FGS drop out of American universities compared to only seven percent of students with parental academic backgrounds (Jehangir, 2010). Moreover, FGS tend to achieve lower grade point averages than CGS (Redford and Hoyer, 2017). In addition, FGS seem to struggle more with their mental health in the university setting. FGS report higher rates of feeling stressed as well as higher levels of depression (Stebleton et al., 2014).

These differences between first- and CGS are commonly assumed to stem from the social capital they acquired from their environment (Soria and Stebleton, 2012). Social capital summarizes the amount of information, resources, and knowledge obtained through social interactions (Robison et al., 2002). This mainly occurs with people one is in close relationships with, such as parents or caregivers (Bourdieu, 1986). Students can rely on this capital to understand which norms and rules are established in the academic context. This conglomerate helps students to navigate their lives in the academic context and make the right educational choices (Pascarella et al., 2004). Students whose parents did not attain tertiary education lack these personal relationships as a resource for guidance (Pascarella et al., 2004; Gofen, 2009; Ives and Castillo-Montoya, 2020). The absence of knowledge and resources on how to navigate the university setting might lead to FGS feeling out of place in academia. Whereas CGS seem to just fit in with the higher educational setting, FGS might struggle more to find their way around (Jehangir, 2010). A possible way for students to overcome obstacles posed by the pathway to an academic degree is to seek support from their instructors during class. However, FGS also differ from CGS regarding the way they interact with their social environment. Although many FGS seek interaction with the faculty, they seem not to obtain it in the same way CGS do. Miyazaki and Janosik (2009) found that FGS ask fewer questions and seek less help from the faculty members in comparison to their peers with an academic parental background. One probable explanation is that FGS avoid interacting with faculty due to concerns over being perceived as incompetent, leading to lower-quality interactions with faculty (Ives and Castillo-Montoya, 2020). Accordingly, FGS may perceive the university setting as less supportive than CGS, which fosters a sense of isolation in the academic context (Spiegler and Bednarek, 2013).

Besides support from faculty, social interaction with peers can also help overcome challenges in the educational setting. Unfortunately, in comparison to CGS, FGS also face unique challenges when connecting with other students (Spiegler and Bednarek, 2013). Studies show that FGS must work significantly more in their spare time to finance their academic education in comparison to peers whose parents attained an academic degree (Spiegler and Bednarek, 2013). In addition, working a side-job seems to obstruct cognitive ability more for FGS than CGS, further impairing academic growth (Pascarella et al., 2004). For FGS, such work obligations also reduced the time they could spend with other students leading to less social interaction and thus to a lower feeling of social belonging with other students (Terenzini et al., 1996).

Yet, social interaction with other students may be particularly beneficial for FGS (Pittman and Richmond, 2007). Studies have shown that FGS can be characterized as prosocial learners as they exhibit not only the desire to learn together with their peers but that their learning is also beneficial to their communities (Pelco et al., 2014; Ives and Castillo-Montoya, 2020). Moreover, Eddy and Hogan (2014) showed that an interdependent approach to learning not only increased the feeling of community amongst students but also minimized the gap in grades between FGS and CGS. Therefore, fostering social interaction in the university setting, which FGS seem to lack, might help to compensate for the lack of social capital in comparison to CGS.

### Consequences of the COVID-19 Pandemic for Students

COVID-19 disrupted the lives of people around the globe in many ways by posing a threat to everyone’s health. Up until November 2021 the deaths of around 5.2 Million people were related to COVID-19, making it one of the most incisive events in recent history (Organization World Health, 2021). Governments were forced to implement restricting measures like curfews, bans of social gatherings, and the mandatory wearing of face masks to mitigate the spread of the virus. Thus, people greatly restricted their personal freedom to keep themselves and others safe. The longer the pandemic lasts, and in the face of limited hope the virus will vanish anytime soon, the more we understand about the psychological implications of these restrictions. Such restrictions likely interfered with basic human psychological needs, such as autonomy and relatedness (Deci and Ryan, 2000).

Students were particularly impacted by the pandemic as educational institutions were shut down (Aristovnik et al., 2020). Firstly, universities faced the sudden and urgent challenge to adapt courses to online learning and struggled with putting a well-grounded eLearning environment into place from 1 day to the next (Oliveira et al., 2021). Due to this unexpected transition, teaching was put on hold or was continued rudimentarily, meaning that interaction between faculty staff and students was reduced to a minimum. In a short time, students went from having a well-structured academic timetable to finding themselves in an uncertain and unpredictable environment. Secondly, for many students, universities are the primary place to meet and connect with their peers. Due to the lockdown measures, students were denied the possibility of meeting their social contacts at or outside of university. Limitations on social interactions might have led to students feeling less integrated with their peers and experiencing less social belonging than those from uninterrupted years. Thus, the closing of the universities impacted not only the educational development of students but their social interaction as well.

These circumstances might have been particularly challenging for FGS (Soria et al., 2020). Lacking interaction with both faculty and peers may harm FGS more than CGS as this might completely remove any social connection to the academic environment. Additionally, many FGS faced the unique challenge of moving back to their family home, finding themselves in an environment unfamiliar with academic study and often unable to effectively provide assistance with any uncertainties related to studying at a tertiary level. As a result, FGS might have felt even more isolated during the lockdown and thus experience even lower social belonging. Thus, when facing challenges in the educational context, their social contacts are not a resource they can rely on in pandemic times. FGS might therefore have perceived the consequences of the pandemic in the education context as more threatening than CGS. The loss of both the structure provided by the university system as well as social contact with their fellow students for support means they might not have had sufficient coping mechanisms at their disposal. Consequently, FGS may have felt more helpless and overwhelmed by the situation than their peers with parental academic background.

### System Justification as a Defensive Strategy

First-generation students may feel more threatened and impacted by the COVID-19 pandemic than their counterparts with a parental academic background. They suffer from missing social interaction with both their peers as well as the faculty. The feeling of isolation may be further fostered by students moving back home to their parents and finding themselves in an environment that might have been unable to provide sufficient support for their academic education. In sum, the pandemic and the resulting consequences might have led FGS, more than CGS, to feel helpless, overwhelmed, and under a great amount of stress.

These circumstances might result in FGS feeling disadvantaged due to their parents’ education. System justification theory (SJT; Jost et al., 2004) takes on the question of how underprivileged individuals can rely on the system they are living in, and what function this reliance serves. Initially, SJT assumed that people are motivated to keep a positive image of themselves at all times (Jost et al., 2004). This motive can occur on different levels. Firstly, people strive to maintain a favorable self-image by seeing themselves as legitimate and valid individuals (ego justification). Secondly, people try to establish a positive image of the group they identify with. Therefore, they try to justify the actions of ingroup members and to maintain a positive image of their respective groups (group justification). The last level of justification refers to the system one is part of. People see the *status quo* of the system as fair, inevitable, and legitimate (Jost et al., 2004).

A major tenet of SJT is disadvantaged individuals can still show justification for the system, nonetheless. The radical form of SJT even proposes that disadvantaged people show more system justification than members of groups favored by the system (Jost et al., 2004). The motivation to defend a system one has a low status in goes against the principle of both ego and group justification and therefore creates cognitive dissonance. This cognitive tension is accompanied by negative psychological states such as anxiety, guilt, and uncertainty (Harding and Sibley, 2013). To reduce these negative states, SJT assumes that disadvantaged people justify the system even more, and therefore accept their underprivileged position (Caricati and Sollami, 2018). System justification can reduce cognitive dissonance and collateral negative states, thereby operates as a defensive strategy (Jost and Hunyady, 2003; Harding and Sibley, 2013). Even as an underprivileged member, defending the system can lead to positive affect as well as increased life satisfaction (Rankin et al., 2009). For instance, in the context of the pandemic, an experiment by Jutzi et al. (2020) showed that the threat salience of COVID-19 led participants to report higher levels of behavioral inhibition and collateral anxiety. This increase in behavioral inhibition was then related to further justifying the political system. Apparently, system justification serves as a defensive strategy to defend against the threat of COVID.

Further research on this counterintuitive phenomenon has brought the aspect of social identity theory (SIT) into play. One tenet of SIT is that people always try to establish a positive self-image, both individually but especially on a collective level (Tajfel and Turner, 1979). Disadvantaged groups are therefore expected to challenge systems that are perceived as illegitimate and unfair. Thus, SIT and SJT propose different approaches for disadvantaged groups facing an unfair system: Whereas SIT proposes an active, challenging role for disadvantaged groups and suggests they exhibit lower levels of system defense, SJT expects underprivileged people to come to terms with their role in the system and even defend it (Caricati and Sollami, 2018).

### System Justification Amongst First-Generation Students

We argue that FGS are disadvantaged in the higher education system and assume FGS pose a vulnerable group in the academic context, especially in threatening situations like the COVID-19 pandemic. System justification would be one possible defense strategy. However, there are several reasons why FGS might justify the system less in comparison to CGS.

First-generation students deviate from other disadvantaged groups as they are actively striving to establish themselves in the unknown educational environment. By striving for a tertiary degree, FGS are challenging their own (comparatively) low hierarchical status in the university system and aiming to change rather than justify their disadvantaged position. Furthermore, in their theoretical paper, Kay and Friesen (2011) claim that system justification should be higher amongst disadvantaged groups that are both dependent on the system and perceive it as inescapable. Laurin et al. (2010) manipulated the ease of transferring universities and afterward presented participants with critical statements about the university. Students who thought switching universities was difficult and thus perceived the system as inescapable were less supportive of the criticism. Consequently, these students reported more system justification than students who assumed they could transfer easily between universities. We argue that FGS may report less justification of the university system than CGS in the wake of the pandemic. Although studies have shown that defending the system can help cope with threat and therefore operates as a defensive strategy (Harding and Sibley, 2013), it is yet to be investigated if system justification works for both FGS and CGS in the same way.

We assume that CGS benefit more from system justification as a defensive strategy than FGS. Due to their parental background CGS perceive the university system as just and can therefore rely on it when facing threats. FGS however, may be less likely to capitalize on system justification as an effective defense.

### Present Research

We assert that lacking social interaction with their peers and faculty as well as feeling misplaced in the higher education setting makes FGS a vulnerable group when facing threats to their educational progress. Due to FGS specific circumstances, such threatening conditions should easily deplete the resources required to counteract negative consequences of threat. The mechanisms of how FGS deviate from their peers with an academic parental background in the way they perceive the burden of the pandemic, have yet to be investigated.

We assume that both the perception of the university as fair and legitimate as well as maintaining fruitful social interactions act as defensive strategies when students are faced with pandemic threat. To explore the different pathways FGS and CGS took while coping with the pandemic, we focused on both students’ perception of the situation as well as their reported ability to act. We assessed whether students perceived the altered learning situation more as a threat or as a challenge. We further explored the impact of three collective defensive strategies, system justification, academic identity, and social belonging, on students’ reported coping with failure in the academic setting as well as their experienced helplessness during the transition to online learning.

We conducted a large-scale online survey to assess coping mechanisms among Austrian students and the differences between FGS and CGS regarding the use of defensive strategies: We first explored the defensive function of system justification for all students independent of their parental academic background. The more students justify the university system and perceive it as fair, the less they should feel helpless, and show better coping with failure and rate the pandemic as less threatening. In a second step, we explored whether FGS and CGS differed regarding the amount of system justification. As argued above, FGS may report lower levels of system justification in comparison to CGS. We then analyzed pathways that students use to cope with the threat to their academic progress. Specifically, we contrasted their use of system justification, academic identity, and social belonging in the form of personal relationships to deal with this threat. Students without parental academic background may rely more on their social belonging in the form of personal relationships than CGS.

## Materials and Methods

Participants were recruited *via* social media, with the data being collected between the start of April and May 2020. Only students enrolled at Austrian universities were allowed to take part, with *N* = 895 completing the online survey presented on LimeSurvey. We excluded 47 participants either because of suspicious and unrealistic response patterns (aspired number of credits during summer term of 60 or more credits) or because of being older than 39 years, which was more than two standard deviations above the mean. Furthermore, we expected that because of different living circumstances, participants older than 39 years would not be comparable to the majority of the student sample, which was in their early twenties. The final sample consisted of 848 participants in the analysis (*M*_age_ = 23.91 years, range 18–35 years, SD_Age_ = 4.04 years; 159 identifying as male, 677 as female and 12 as diverse; 644 Austrian, 142 German, 62 other/no nationality indicated).

Before partaking, participants gave informed consent consistent with the declaration of Helsinki with instructions and were informed they could leave the survey at any point. To determine the first-generation status of the participants, we asked for the highest educational degree of both parents. Academic parental background was assumed when at least one parent attained a bachelor’s or higher degree. According to this classification, 515 (60.73%) participants were classified as FGS and 333 (39.27%) participants were categorized as CGS. The present study was part of a larger research project and we report only the measurements pertinent to the current investigation.

### Perceived COVID-19 Threat for Academic Progression

We assessed the perceived threat of the pandemic on individual academic progress with the item ‘‘I am afraid of not being able to complete enough ECTS^1^ this summer semester 2020 due to the COVID-19 pandemic.” Participants could respond on a six-point Likert scale ranging from “disagree completely” to “agree completely” (*M* = 3.60, SD = 1.96). Despite being a single-item scale, the perceived threat of the pandemic on individual academic progress seems to be a valid measure showing a strong correlation with the behavioral proxy of uncertain credits, *r*(846) = 0.46, *p* < 0.001, due to the pandemic. In addition, perceived threat of the pandemic on individual academic progress correlates with additionally collected outcomes such as satisfaction, *r*(846) = −0.38, *p* < 0.001, and wellbeing of the students, *r*(846) = −0.36, *p* < 0.001, during lockdown. All three measures used for validation were each assessed using single-item scales (see Supplementary Materials 1.1.1–1.1.3 for exact item descriptions).

### System Justification

We measured system justification with an adaptation of the system justification scale from Kay and Jost (2003) to fit the university setting (see Supplementary Material 1.2). Participants answered seven items on a six-point Likert scale ranging from “completely disagree” to “completely agree” (e.g., “Current teaching at my university is structured so that students generally get what they deserve;” Cronbach’s alpha: α = 0.91). CFA indicated a good fit for a single factor solution, χ^2^ (6) = 7.786, RMSEA = 0.019, SRMR = 0.008, CFI > 0.999 (Hu and Bentler, 1999). The estimated latent variable was used for all subsequent analyses. Latent variables and the resulting factor models are a covariation-based method to investigate and measure unobservable constructs, e.g., system justification (Borsboom et al., 2003). In addition, the fit of these factor models indicates to what extent the data supports the suggested underlying latent variable structure, making it a more comprehensive approach than mean score scales.

### Academic Identity

Academic identity was measured using a newly developed scale (see Supplementary Material 1.3). The scale consisted of eight items (e.g., “I can identify well with my studies”). Participants responded to the statements with a six-point Likert scale ranging from “completely disagree” to “completely agree.” Cronbach’s alpha was good, α = 0.84. We estimated the latent variable using a CFA, which showed a good fit for a single factor solution, χ^2^ (5) = 6.479, RMSEA = 0.019, SRMR = 0.010, CFI = 0.999. This latent variable was used for all following analyses.

### Social Belonging

We measured social belonging focusing on personal relationships using a novel scale (e.g., “I already have many good contacts with the other students in my department,” see Supplementary Material 1.4). Participants responded to five statements on a six-point Likert scale ranging from “completely disagree” to “completely agree.” Cronbach’s alpha indicated good reliability, α = 0.83. CFA indicated a good fit of a single factor solution, which we used for all subsequent analyses, χ^2^ (1) = 0.049, RMSEA < 0.001, SRMR = 0.001, CFI > 0.999.

### Helplessness

The students’ perceived helplessness regarding learning digitally was assessed with the subscale *Amotivation* of the *Situational Motivation Scale* (Lonsdale et al., 2011; see Supplementary Material 1.5). We presented four items which were answered on a ten-point Likert scale ranging from “Not true at all” to “completely true.” The items were reformulated to fit the digital learning context e.g.: “I don’t know: I can’t see what digital learning brings me.” Cronbach’s alpha was very good, α = 0.90, and CFA supported a single factor solution, χ^2^ (1) = 4.155, RMSEA = 0.061, SRMR = 0.006, CFI = 0.999. The estimated latent variable was used for the following analyses.

### Threat Versus Challenge

The perception of the learning situation during the shutdown of educational institutions as a threat and as a challenge were measured using an eight-item scale, adapted from Drach-Zahavy and Erez (2002). The statements, respectively, four for threat and challenge, were adapted for the students’ learning situation (see Supplementary Material 1.6). Example items for threat and challenge are “I worry that I lack the skills to handle the situation” and “The situation gives me the opportunity to expand my skills.” Cronbach’s alpha was good for both the threat and challenge scale, α = 0.85 and α = 0.80. We conducted a CFA for threat, χ^2^ (1) = 6.419, RMSEA = 0.080, SRMR = 0.013, CFI = 0.996, and challenge, χ^2^ (1) = 0.608, RMSEA < 0.001, SRMR = 0.005, CFI > 0.999, respectively. Both confirmatory factor analyses indicated an acceptable fit and the estimated latent variables were used in the subsequent analyses.

### Maladaptive Coping With Failure

To assess maladaptive coping with failure we used the German subscale “Coping with failure” of the SSI-K3 (Kuhl and Fuhrmann, 1998; see Supplementary Material 1.7). Participants answered four statements on an eleven-point Likert scale ranging from “not at all” to “exceptionally” (e.g., “When something bad has happened, it takes me a long time to focus on something else.”). Cronbach’s alpha indicated good reliability, α = 0.87. CFA implied a good fit for a single factor solution, which was further used for following analyses, χ^2^ (1) = 0.624, RMSEA < 0.001, SRMR = 0.003, CFI > 0.999.

### Data Analysis

To explore which pathways FGS and CGS use when faced with threat we calculated structural equation models using lavaan 0.6-7 in R 4.0.2 (Rosseel, 2012; R Core Team, 2021). Structural equation model parameters were estimated *via* maximum likelihood method with 5,000 bootstraps. We opted to use structural equation modeling in contrast to ordinary regression methods for several reasons. Firstly, structural equation modeling does not only estimate the relationships between dependent and independent variables, it also incorporates a confirmatory factor analysis (Lei and Wu, 2007). Therefore, this statistical method allows for investigating how well indicators load on the construct in contrast to calculating mean scores. This integral part is missing in conventional regression-based approaches. Moreover, structural equation modeling also takes measurement errors into account and thus provides a corrected estimation of coefficients (Anderson and Gerbing, 1988; Lei and Wu, 2007). In comparison to conventional regression-based analysis, structural equation modeling offers the possibility to estimate the influence of predictors on multiple dependent variables and therefore facilitates the testing of complex relationships between variables (Biddle and Marlin, 1987). Lastly, structural equation modeling allows for correlations between indicators and variables which represents the data collected in the field more appropriately (Lei and Wu, 2007). For correlations between the tested variables, see Table 1. To test for differences in system justification between FGS and CGS we conducted an independent students’ *t*-test. If pathways from defensive strategies on dependent variables were significant for FGS but not CGS, or vice versa, we used moderation analysis to investigate the influence of first-generation student status. The corresponding defensive strategy, first-generation student status, and its interaction functioned as predictors.

**TABLE 1:** Means, standard deviations, and correlations of tested variables.

Variable	*M*	SD	1	2	3	4	5	6	7
1. Threat of COVID-19	3.60	1.96							
2. System justification	3.60	1.26	−0.41***						
3. Academic identity	4.56	1.02	−0.15***	0.45***					
4. Social belonging	4.37	1.13	−0.19***	0.19***	0.35***				
5. Helplessness	3.89	2.50	0.21***	−0.36***	−0.23***	−0.08*			
6. Digital learning perceived as challenge	3.06	1.39	−0.26***	0.45***	0.24***	0.06	−0.41***		
7. Digital learning perceived as threat	2.70	1.35	0.40***	−0.31***	−0.20***	−0.19***	0.39***	−0.39***	
8. Maladaptive coping with failure	5.15	2.53	0.12***	−0.17***	−0.18***	−0.13***	0.18***	−0.17***	0.44***

*p < 0. 05; ***p < 0.001.

## Results

### The Defensive Role of System Justification

We first explored the use of system justification as a defensive strategy for all students, independent of their parents’ academic background and investigated the relationship between the perceived threat of the pandemic obstructing academic progress and system justification. Assuming system justification is an effective means to cope with threat, it should be related to less helplessness, less maladaptive coping with failure, and perceiving the altered learning situation less as a threat and more as a challenge.

**FIGURE 1:**
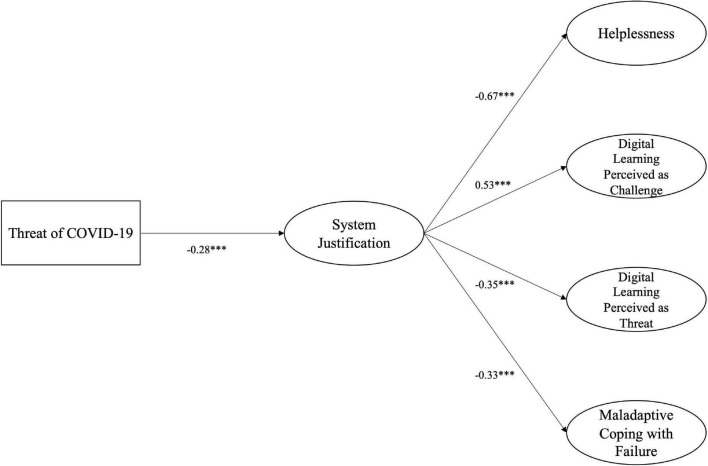
Structural Equation model of the defensive function of system justification. *N* = 848, CFI = 0.943; RMSEA = 0.060; SRMR = 0.080. The model shows the unstandardized regression coefficients. Only significant paths are depicted. For paths, see Supplementary Table 2. ***p < 0.001.

SRMR indicated an acceptable model fit (SRMR = 0.080), but other indicators showed weaker fit (CFI = 0.943; RMSEA = 0.060). The model and unstandardized regression coefficients are depicted in Figure 1. Perceived COVID-19 threat for academic progression was significantly negatively related to system justification, meaning that students who feared a lack of academic progress due to the pandemic also justified the university system less, *b* = −0.28, SE = 0.02, *p* < 0.001, 95% CI (−0.33, −0.23). We observed a defensive function of system justification for helplessness, *b* = −0.67, SE = 0.06, *p* < 0.001, 95% CI (−0.80, −0.54), perception of the learning circumstances as a threat, *b* = −0.35, SE = 0.04, *p* < 0.001, 95% CI (−0.44, −0.27), and maladaptive coping with failure, *b* = −0.33, *SE* = 0.07, *p* < 0.001, 95% CI (−0.47, −0.18). System justification was also associated with higher levels of perceiving the current education situation as challenging, *b* = 0.53, SE = 0.04, *p* < 0.001, 95% CI (0.45, 0.61).

### Differences in System Justification Regarding First-Generation Student Status

We first extracted the latent variable system justification from the structural equation model above to compare first- and CGS. An independent student’s *t*-test indicated a small but significant difference between FGS and CGS, *t*(731.19) = 2.29, *p* = 0.022, *d* = 0.16. FGS (*M* = −0.08, SD = 1.23) justified the university system in the wake of the pandemic significantly less than CGS (*M* = 0.12, SD = 1.18).

**FIGURE 2:**
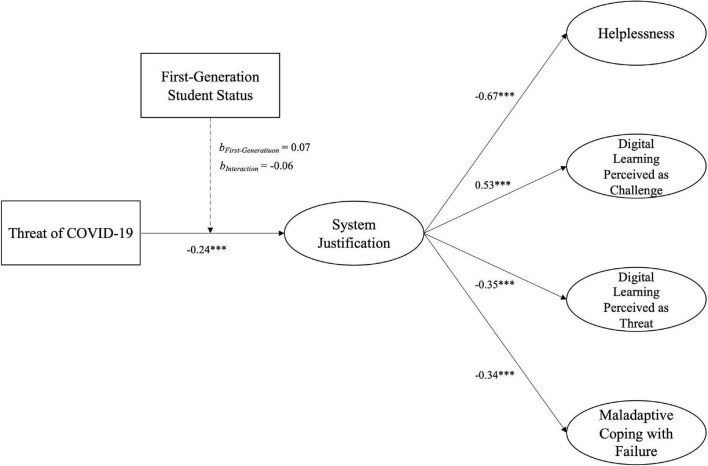
Structural Equation model of the defensive function of system justification including first-generation student status. *N* = 848, CFI = 0.943; RMSEA = 0.055; SRMR = 0.077. The model shows the unstandardized regression coefficients. Only significant paths are depicted. For paths, see Supplementary Table 3. ***p < 0.001.

Further, investigating whether first-generation status had an impact on the relationship between threat and system justification, we added the parental academic background as a moderator on the path in the structural equation model. The parental academic background did not influence the association between COVID-19 threat and system justification, main effect FGS status, *b* = 0.07, SE = 0.18, *p* = 0.683, 95% CI (−0.27, 0.43), FGS status × threat interaction, *b* = −0.06, SE = 0.05, *p* = 0.204, 95% CI (−0.15, 0.03), and the influence of threat on system justification remained significant when including FGS status into the model, *b* = −0.24, SE = 0.04, *p* < 0.001, 95% CI (−0.32, −0.17) (Figure 2 for direct effects of first-generation student status on dependent variables see Supplementary Table 3).

However, it has yet to be investigated whether system justification and other defensive strategies are associated with the students’ perception of the altered learning situation in the same way for FGS and CGS. To compare the different pathways both student groups may take, we conducted separate analyses for FGS and CGS.

### Continuous-Generation Students

**FIGURE 3:**
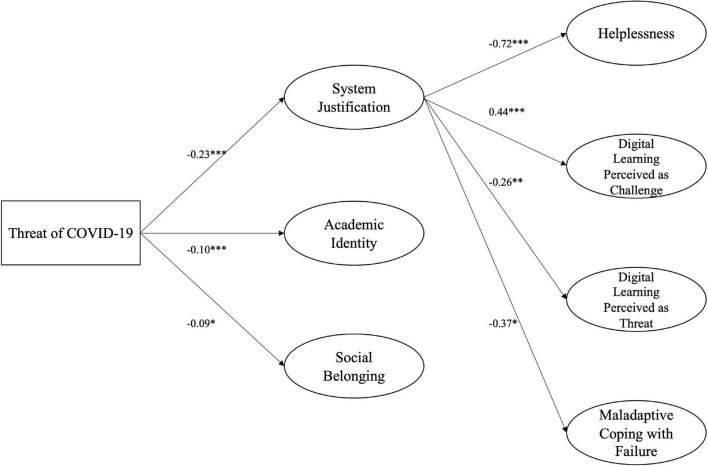
Structural Equation model of defensive strategies for CGS. *N* = 333, CFI = 0.885; RMSEA = 0.065; SRMR = 0.126. The model shows the unstandardized regression coefficients. Only significant paths are depicted. For paths, see Supplementary Table 4. *p < 0.05; **p < 0.01; ***p < 0.001.

We investigated whether FGS and CGS differ regarding their use of system related defenses. Therefore, we tested the model above for FGS and CGS separately. For CGS, model fit was somewhat weak (CFI = 0.885; RMSEA = 0.065; SRMR = 0.126). Threat was negatively associated with all three defenses. For CGS, higher levels of threat led to significantly less system justification, *b* = −0.23, SE = 0.04, *p* < 0.001, 95% CI (−0.31, −0.16), academic identity, *b* = −0.10, SE = 0.03, *p* < 0.001, 95% CI (−0.14, −0.05), and social belonging, *b* = −0.09, SE = 0.04, *p* = 0.029, 95% CI (−0.16, −0.01). However, only system justification showed any relation to the dependent variables. The more CGS justified the system, the less helplessness, *b* = −0.72, SE = 0.14, *p* < 0.001, 95% CI (−0.99, −0.46), lower levels of threat perception of the learning situation, *b* = −0.26, SE = 0.08, *p* = 0.001, 95% CI (−0.43, −0.11), and maladaptive coping with failure they reported, *b* = −0.37, SE = 0.15, *p* = 0.013, 95% CI (−0.67, −0.09). Furthermore, a significant positive relationship between system justification and perception as a challenge was observed, *b* = 0.44, SE = 0.08, *p* < 0.001, 95% CI (0.28, 0.60). Neither academic identity nor social belonging had a significant impact on the dependent variables (see Figure 3).

### First-Generation Students

**FIGURE 4:**
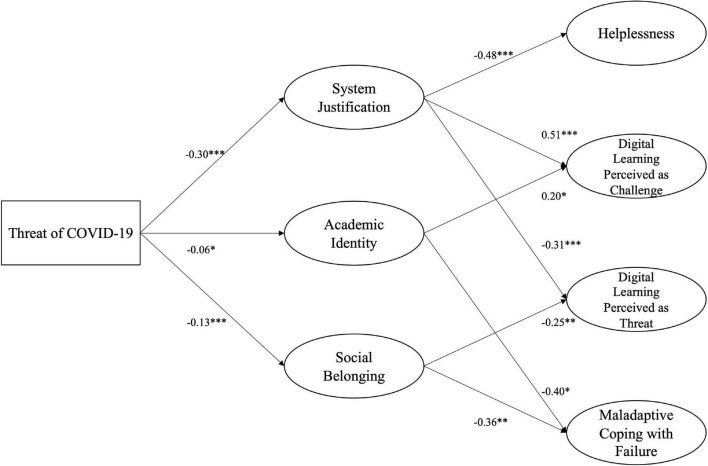
Structural Equation model of defensive strategies for FGS. *N* = 515, CFI = 0.881; RMSEA = 0.069; SRMR = 0.141 The model shows the unstandardized regression coefficients. Only significant paths are depicted. For paths, see Supplementary Table 5. *p < 0.05; **p < 0.01; ***p < 0.001.

For FGS, the model delivered a somewhat weak fit (CFI = 0.881; RMSEA = 0.069; SRMR = 0.141). Perceived COVID-19 Threat for academic progression was significantly related to system justification, *b* = −0.30, SE = 0.03, *p* < 0.001, 95% CI (−0.36, −0.25), academic identity, *b* = −0.06, SE = 0.03, *p* = 0.020, 95% CI (−0.11, −0.01), and social belonging, *b* = −0.13, SE = 0.03, *p* < 0.001, 95% CI (−0.19, −0.09). When FGS justified the university system they significantly reported less helplessness, *b* = −0.48, SE = 0.09, *p* < 0.001, 95% CI (−0.67, −0.32) and perception of the educational situation as a threat, *b* = −0.31, *SE* = 0.06, *p* < 0.001, 95% CI (−0.43, −0.21), but more perception as a challenge, *b* = 0.52, SE = 0.06, *p* < 0.001, 95% CI (0.40, 0.63). Academic identity led to less maladaptive coping with failure, *b* = −0.40, SE = 0.17, *p* = 0.019, 95% CI (−0.70, −0.05), but higher levels of perception as a challenge, *b* = 0.20, SE = 0.10, *p* = 0.035, 95% CI (−0.03, 0.34). Social belonging was significantly negatively related to the perception of the learning situation as a threat, *b* = −0.25, SE = 0.09, *p* = 0.006, 95% CI (−0.45, −0.09), and maladaptive coping with failure, *b* = −0.36, SE = 0.14, *p* = 0.009, 95% CI (−0.65, −0.11) (see Figure 4). Comparing both structural equation models, it suggests that FGS, in comparison to CGS, not only use system justification as a defensive strategy but also rely on their academic identity as well as their connection to peers and faculty staff.

### Differences in Pathways for First-Generation Students and Continuous-Generation Students

There were a total of five paths that were either significant for FGS but not CGS, or vice versa: (1) System justification on maladaptive coping with failure, (2) academic identity on perception of the learning situation as a challenge, (3) academic identity on maladaptive coping with failure, (4) social belonging on perception of the educational circumstances as a threat, and (5) social belonging on maladaptive coping with failure. To investigate whether first-generation student status had an influence on these pathways, we calculated moderation analyses.

To this end, we extracted all latent variables estimated by the structural equation model above and then conducted a multiple regression on the dependent variables (see Supplementary Tables 6–10). The corresponding collective defensive strategy, first-generation student status, and the interaction functioned as regressors. We found a significant moderation for social belonging and first-generation status on perception of the altered learning situation as a threat, *b* = −0.21, *p* = 0.015. Thus, FGS benefited more from social belonging in the form of close relationships than CGS and thus reported lower levels of threat perception. Moreover, FGS status moderated the association of social belonging on maladaptive coping with failure, *b* = −0.32, *p* = 0.043. Feeling socially integrated therefore had a significantly stronger impact for FGS than for CGS. In addition, results showed a significant moderation effect for the regression from academic identity and first-generation status on perception as challenge, *b* = 0.22, *p* = 0.043, with a significant main effect of academic identity, *b* = 0.23, *p* = 0.006. FGS interpreted the situation more as a challenge in comparison to CGS when reporting higher academic identity.

## Discussion

This large-scale study explored how first-generation and CGS differed in how they used system justification and social belonging as defensive strategies to cope with the threat of the COVID-19 pandemic to their academic progress. Firstly, we observed that the perceived threat of COVID-19 on academic progress was negatively related to system justification, such that students with higher threat perceptions also defended the university system less. System justification was used as a defensive strategy during the shutdowns by both CGS and FGS. Independent of their parental academic background, students who defended the academic system more reported less helplessness, less maladaptive coping, and perceived their altered learning situation as a challenge rather than a threat. However, we found that FGS reported significantly less system justification in comparison to CGS. Apparently, FGS do not fully exploit the defensive function of system justification. Using structural equation models, we explored the pathways FGS and CGS relied on when faced with this threat to their academic progress. CGS only used system justification as a defensive strategy to cope with the threat. CGS who justified the system more felt less helpless, perceived the learning situation as a challenge rather than a threat, and reported less maladaptive coping with failure. Academic identity and social belonging were not significantly related to these threat responses. However, FGS relied on all three defensive strategies when coping with threat. Similar to CGS, they benefited when perceiving the university system as just; however, academic identity and social belonging in the form of personal relationships helped them cope more effectively with the threat to their academic progress.

### Contribution to System Justification Theory

We explored the role of SJT under the acute threat of the COVID 19 pandemic. The observed higher levels of FGS are in contrast to classic system justification research (Jost et al., 2004). According to classic research, FGS should be a disadvantaged group due to their parental academic background, and defending the system should help them perceive their underprivileged position as more legitimate. While in our sample FGS and CGS indeed both benefited from this defensive function of system justification (i.e., reduced feelings of helplessness and threat as well as better coping with failure) FGS justified the university system not more but actually less than CGS. Although contrary to initial SJT research, our exploratory findings are in line with Caricati and Sollami (2018) who reported similar patterns when evaluating system justifications amongst disadvantaged people.

A possible explanation for this phenomenon could be that our sample of Austrian students may differ from other evidence regarding system justification amongst disadvantaged individuals. Extensive research on system justification has been conducted for groups who are in an underprivileged position in society in general due to their ethnic or socioeconomic background (Hässler et al., 2019; Negrete and Hurd, 2020; Essien et al., 2021). Although FGS are often part of other marginalized groups, for most FGS this disadvantaged position only applies to the isolated area of the tertiary education system. Moreover, representing approximately 60% of all students in Austria, FGS may be disadvantaged but are not the minority. In sum, our findings point to the need for further systematic research on the role of system justification in coping with threat, both for advantaged and disadvantaged groups.

When interpreting the present results with regard to previous research on FGS, it is important to take into account that most of the research on FGS has been conducted in the United States (Spiegler and Bednarek, 2013). Whereas in the United States, FGS account for only a third of all students, FGS in Austria are even in the majority with approximately 60%, as in our sample (Ives and Castillo-Montoya, 2020; Institut für Höhere Studien, 2021). In Austria, FGS might experience unique challenges on their educational pathways due to their parental academic background, but this applies to the majority of students. Therefore, connecting with peers might be particularly beneficial for FGS in Austria as many students can relate to their shared situation. In our sample, FGS are disadvantaged (university-related social capital) but not marginalized. In the United States, they are most likely both. Another difference to the United States is the importance of tertiary education. Different to central Europe, the likelihood of attaining a well-paid job without a post-secondary degree is low (Greiner et al., 2004; Autor et al., 2008). This might lead to lower standards of living and resulting challenges. Therefore, the difference between FGS and CGS in the United States may carry more weight than it does in central European countries.

### Contributing to Understanding Continuous-Generation Students and First-Generation Students at University

Our analyses indicated that CGS solely relied on system justification to cope with the pandemic threat. Academic identity and social belonging were not related to the perception and experience of CGS. Therefore, a university system perceived as just and legitimate by CGS can operate as a buffer against the burdening consequences of the pandemic. Close relationships with peers or a strong academic identity were not as important for students with parental academic background.

In contrast, our analyses showed that FGS rely on a variety of defensive strategies when faced with threat. Similar to CGS, FGS benefited from perceiving the university system as fair and legitimate. Yet, the importance of academic identity and social belonging set them apart from CGS. To effectively cope with the threat of the pandemic to their academic progress they relied heavily on concrete relationships with peers and faculty. Apparently, FGS benefit the most when they can trust and lean on a conglomerate of both abstract strategies, such as system justification as well as concrete defenses in the form of personal relationships. This is good news as FGS may have more strategies at their disposal and are not as dependent on system justification as CGS.

Abstract and concrete defensive strategies differ in the way they are available to individuals. Abstract strategies such as system justification are always at the individual’s disposal since they are only mentally constructed. Furthermore, this resource can not be depleted and thus individuals can endlessly rely on it. In contrast, concrete defensive strategies such as social belonging require an external basis. Social belonging can hardly be established without making and maintaining personal relationships. In addition, these external resources, such as friendships, can be depleted, meaning individuals can not rely on them infinitely. Therefore, it is important for both CGS and, more especially, FGS to make use of abstract strategies in addition to concrete strategies.

### Limitations and Future Research

Some limitations of our research should be noted. Although we draw on a large sample during a crucial time, we used a cross-sectional correlational design that precludes strong causal conclusions. In our model, we proposed that students were first faced with the threat of the pandemic to their academic progress. Defensive strategies such as system justification or social belonging then helped them cope to feel less helpless or interpret the altered situation more as a challenge. However, it is also arguable that collective strategies come first. Students could feel less integrated with others and do not perceive the university system as just and legitimate in the first place, leading to them feeling helpless or cope less well with failure. These constraints could then be positively associated with perceived threat to their academic progress. We tested this approach by conducting another structural equation model (see Supplementary Table 11). The model showed a comparable fit to our initially suggested approach for FGS and CGS. Another option to be considered is that the perceived threat on academic progress, defensive strategies, and the feeling of helplessness for example could reinforce each other. High levels of perceived threat could lead to low system justification and then to increased helplessness, which then further fosters the perceived threat. This vicious circle makes it difficult to determine a precise causal model without experimental manipulation. In summary, it is yet to be investigated which causal relationships occur for both FGS and CGS following the threats to academic progress due to the COVID-19 pandemic. Therefore, we would encourage further research to focus on exploring the possible causal relationship. For instance, experimental manipulation could provide insight into whether FGS and CGS differ in the way they establish and capitalize on system justification as a defensive strategy.

Furthermore, this article presents a cross-sectional study. Thus, only statements about the students’ situation at the beginning of the pandemic can be derived. Taking into account the rapidly and ever-changing dynamics of the pandemic it can be assumed that the pathways students use to cope also change. With the pandemic still ongoing, institutions have been able to make adjustments to improve the online learning environment. This could be especially beneficial to FGS as an increase in interactive tools allows for more social contact with their peers. Thus, it would be insightful to compare the students’ situation and their strategies at the beginning of the pandemic to the current circumstances. In addition, we used social media to recruit participants for our study. Although the composition of our sample represents a large part of the target population, this method of collecting data could pose a limitation to the generalizability of our findings. We thus would encourage future research to strive for fully representative samples.

The somewhat poor fit of some of the structural equation models has to be taken into account when interpreting the results. Yet, this methodological approach to analyzing the data seems appropriate for several reasons. Firstly, it also allows us to test complex relationships between variables which assume occur in the field as well. More importantly, structural equation modeling uses latent variables for calculation and therefore is a more conservative analysis than other conventional regression-based models. The poor fit could also potentially stem from the adequate but not stratified sample.

We found that disadvantaged individuals, in our case FGS, justified the university system less than privileged students. This observation challenges a large body of research on SJT and is therefore in need of further replication. Future research could lead to a more balanced view of the role system justification plays for disadvantaged individuals. In particular, the defensive function of system justification when threatened could be further explored. Moreover, this study focuses on defensive strategies when faced with the threat of COVID-19 pandemic solely in tertiary education. To further understand the role of these strategies it would be interesting to investigate the function of system justification and social belonging in the younger population in general. Are these defensive strategies bound to the university system or are these opportunities to cope with the threat of COVID-19 on educational progress transferrable to primary or secondary schools?

The different pathways FGS and CGS use give an insight into how to support disadvantaged groups such as FGS in threatening situations. With FGS being heavily reliant on concrete personal relationships to cope effectively with the altered learning situation, educational institutions should foster possibilities for students to connect. This could be accomplished by establishing more interactive courses and using innovative digital tools through which personal contact can be easily increased. Furthermore, institutions can focus on setting up or maintaining extracurricular activities even in pandemic times to encourage these personal relationships. That could lead to higher social belonging among FGS and CGS, making it a reliable resource for coping with threats to their academic progress. Fostering social belonging through interventions may be particularly beneficial for disadvantaged students, in our case FGS. Walton and Cohen (2007, 2011) showed social belonging interventions improved academic outcomes, particularly amongst disadvantaged and minority students, reducing the academic achievement gap to privileged students. As FGS seem to rely on social belonging as a defensive strategy when threatened, they could particularly profit from these interventions.

Besides interventions or adaptations by the university, students can also make changes to their own behavior to help them cope with the COVID-19 pandemic. Recent research indicates that planning is an effective way for groups and teams to navigate through pandemic times. In particular, collective implementation intentions, or We-if-then planning, seems to be beneficial when under threat (Thürmer et al., 2013, 2020, 2021; Wieber et al., 2015). By planning collectively (e.g., maintaining or establishing learning groups) students could attain their goal of making academic progress more effectively.

## Conclusion

In conclusion, this study explores how students use defensive strategies such as system justification and social belonging to cope with the threat of the COVID-19 pandemic to academic progress. We observed that the parental academic background of students played an important role in which of these strategies students relied on. CGS only benefited from abstract defenses, such as justifying the university system, whereas FGS relied on a conglomerate of both abstract and concrete strategies, like personal relationships. We hope that our research will contribute to helping all students succeed and reach their full potential.

## Data Availability Statement

The raw data supporting the conclusions of this article will be made available by the authors, without undue reservation.

## Ethics Statement

The studies involving human participants were reviewed and approved by the Ethikkommission Universität Salzburg. The patients/participants provided their written informed consent to participate in this study.

## Author Contributions

All authors listed have made a substantial, direct, and intellectual contribution to the work, and approved it for publication.

## Conflict of Interest

The authors declare that the research was conducted in the absence of any commercial or financial relationships that could be construed as a potential conflict of interest.

## Publisher’s Note

All claims expressed in this article are solely those of the authors and do not necessarily represent those of their affiliated organizations, or those of the publisher, the editors and the reviewers. Any product that may be evaluated in this article, or claim that may be made by its manufacturer, is not guaranteed or endorsed by the publisher.

## Footnotes

1 ECTS stands for “European Credit Transfer and Accumulation System.” One ECTS is equivalent to 25 h of workload. Subsequently the term “credits” will be used to ensure comprehensibility (Bundesministerium für Bildung Wissenschaft und Forschung, 2021; European Commission, 2021).
