# Supplementary material for: Exploring Higher Education Pathways for Coping With the Threat of COVID-19: Does Parental Academic Background Matter?
Source: Front Psychol. 2022 Jan 7;12:768334. doi: 10.3389/fpsyg.2021.768334 (PMC8776704; doi:10.3389/fpsyg.2021.768334)
Supplement: Supplementary file 1 [file Data_Sheet_1.pdf]

Supplementary Material

1 Measures[1]

1.1 Validation Items for Perceived COVID-19 Threat for academic progression

1.1.1 Uncertain ECTS

- For how many of your originally planned ECTS is it currently unclear (due to the COVID-19 situation) to what extent you will actually be able to complete them?

1.1.2 Satisfaction

- How satisfied are you with the current learning situation?

1.1.3 Wellbeing

- How comfortable are you with the current learning situation?

1.2 System Justification

- Currently, I think the teaching at my university is fair.
- In this crisis, teaching at my university is working as it should.
- The current teaching at my university should be radically restructured. (-)
- My university is the best university to study now.
- Most of the measures taken are truly for the benefit of students.
- With these measures, everyone has a fair chance to successfully continue their studies.
- Current teaching at my university is structured so that students generally get what they deserve.

1.3 Academic Identity

- I can identify well with my studies.
- I like being a student at this university.
- I have trouble navigating the academic environment. (-)
- I would recommend my studies to others.
- I often feel like I don't really belong at the university. (-)
- My expectations of my studies at this university were fully met.
- Sometimes I wonder if studying is right for me. (-)

1.4 Social Belonging

- I (already) have a lot of good contacts with the instructors at my department.
- I already have many good contacts with the other students at my department.
- I have already become very accustomed to studying.
- I have (already) made friends at university.

---

[1] All Items were presented in German.

- I feel (already) in good hands in my studies

1.5 Helplessness

- There are good reasons for digital learning, but I personally don't see any.
- I'm learning digital, but I don't know if it's worth it.
- I don't know: I can't see what digital learning brings me.
- I'm learning digital, but I'm not sure it's a good thing to pursue.

1.6 Threat versus Challenge

1.6.1 Perception of the Learning Situation During the Shutdown of Educational Institutions as a Threat

- The situation is a threat to me.
- I'm worried that the situation might expose my weaknesses.
- On the whole, it seems to me that I cannot handle the situation.
- I worry that I lack the skills to handle the situation.

1.6.2 Perception of the Learning Situation During the Shutdown of Educational Institutions as a Challenge

- The situation gives me the opportunity to expand my skills.
- The situation offers me the opportunity to overcome obstacles.
- The situation represents for me a way to strengthen my self-esteem.
- In general, I think I can handle the situation.

1.7 Maladaptive Coping With Failure

- After unpleasant experiences, I often can't get out of ruminating for quite some time.
- When something bad has happened, it takes me a long time to focus on something else.
- When I get into a bad mood, it's very difficult to get out of it.
- It's hard to get rid of worrying thoughts once they're there.

2 Structural Equation Models

Table 2

Structural Equation Model: The palliative role of system justification

Regression	*b*	*SE*	*z*-value	*p*-value	95% CI
Threat COVID-19 → System Justification	-0.28	0.02	-12.09	< .001	[-0.33, -0.23]
System Justification → Helplessness	-0.67	0.06	-10.38	< .001	[-0.80, -0.54]
System Justification → Digital Learning Perceived as Challenge	0.53	0.04	12.93	< .001	[0.45, 0.61]
System Justification → Digital Learning Perceived as Threat	-0.35	0.04	-8.59	< .001	[-0.44, -0.27]
System Justification → Maladaptive Coping with Failure	- 0.33	0.07	-4.58	< .001	[-0.47, -0.18]

Note. Regression weights and 95% confidence intervals, based on 5,000 bootstrap samples. *b*-values indicate the unstandardized regression coefficients

Table 3

Structural Equation Model: Differences in system justification regarding first-generation student status[2]

**Regression**	***b***	***SE***	***z*-value**	***p*-value**	**95% CI**
Threat COVID-19 → System Justification	-0.24	0.04	-6.73	< .001	[-0.32, -0.17]
First-Generation Student Status → System Justification	0.07	0.18	0.41	0.683	[-0.27, 0.43]
Threat COVID-19 x First-Generation Student Status → System Justification	-0.06	0.05	-1.27	0.204	[-.15, 0.03]
System Justification → Helplessness	-0.67	0.06	-10.45	< .001	[-0.80, -0.54]
System Justification → Digital Learning Perceived as Challenge	0.53	0.04	12.96	< .001	[0.45, 0.61]
System Justification → Digital Learning Perceived as Threat	-0.35	0.04	-8.70	< .001	[-0.43, -0.27]

[2] Direct effects of first-generation student status on dependent variables

Helplessness: $F(1,846) = 8.43$, $b = 0.40$, $p = .004$

Digital Learning Perceived as Challenge: $F(1,846) = 0.10$, $b = -0.03$, $p = .758$

Digital Learning Perceived as Threat: $F(1,846) = 0.57$, $b = 0.06$, $p = .451$

Maladaptive Coping with Failure: $F(1,846) = 0.05$, $b = -0.03$, $p = .825$

System Justification → Maladaptive Coping with Failure	- 0.34	0.07	-4.57	< .001	[-0.46, -0.19]

Note. Regression weights and 95% confidence intervals, based on 5,000 bootstrap samples. *b*-values indicate the unstandardized regression coefficients

Table 4

Structural Equation Model: Continuous-generation students

**Regression**	***b***	***SE***	***z*-value**	***p*-value**	**95% CI**
Threat COVID-19 → System Justification	-0.23	0.04	-6.29	< .001	[-0.31, -0.16]
Threat COVID-19 → Academic Identity	-0.10	0.03	-3.86	< .001	[-0.14, -0.05]
Threat COVID-19 → Social Belonging	-0.09	0.04	-2.18	.029	[-0.16, -0.01]
System Justification → Helplessness	-0.72	0.14	-5.32	< .001	[-0.99, -0.46]
Academic identity → Helplessness	-0.38	0.22	-1.75	.080	[-0.81, 0.04]
Social Belonging → Helplessness	0.26	0.17	1.55	.122	[-0.05, 0.61]
System Justification → Digital Learning Perceived as Challenge	0.44	0.08	5.34	< .001	[0.28, 0.60]
Academic identity → Digital Learning Perceived as Challenge	0.05	0.14	0.38	.703	[-0.23, 0.31]
Social Belonging → Digital Learning Perceived as Challenge	-0.15	0.11	-1.36	.173	[-0.38, 0.05]

System Justification → Digital Learning Perceived as Threat	-0.26	0.08	-3.20	.001	[-0.43, -0.11]
Academic identity → Digital Learning Perceived as Threat	-0.14	0.14	-1.06	.290	[-0.40, 0.13]
Social Belonging → Digital Learning Perceived as Threat	-0.03	0.10	-0.34	.735	[-0.23, 0.15]
System Justification → Maladaptive Coping with Failure	-0.37	0.15	-2.47	.013	[-0.67, -0.09]
Academic identity → Maladaptive Coping with Failure	-0.17	0.23	-0.75	.453	[-0.64, 0.26]
Social Belonging → Maladaptive Coping with Failure	0.00	0.18	0.01	.989	[-0.37, 0.35]

Note. Regression weights and 95% confidence intervals, based on 5,000 bootstrap samples. *b*-values indicate the unstandardized regression coefficients

Table 5

Structural Equation Model: First-generation students

Regression	*b*	*SE*	*z*-value	*p*-value	95% CI
Threat COVID-19 → System Justification	-0.30	0.03	-10.13	< .001	[-0.36, -0.25]
Threat COVID-19 → Academic Identity	-0.06	0.03	-2.32	.020	[-0.11, -0.01]
Threat COVID-19 → Social Belonging	-0.13	0.03	-5.00	< .001	[-0.19, -0.08]
System Justification → Helplessness	-0.48	0.09	-5.52	< .001	[-0.67, -0.32]
Academic identity → Helplessness	-0.29	0.16	-1.82	.068	[-0.54, 0.10]
Social Belonging → Helplessness	-0.08	0.14	-0.57	.571	[-0.36, 0.18]
System Justification → Digital Learning Perceived as Challenge	0.51	0.06	8.73	< .001	[0.40, 0.63]
Academic identity → Digital Learning Perceived as Challenge	0.20	0.10	2.12	.035	[-0.03, 0.34]
Social Belonging → Digital Learning Perceived as Challenge	0.00	0.09	0.04	.969	[-0.16, 0.19]

System Justification → Digital Learning Perceived as Threat	-0.31	0.05	-5.74	< .001	[-0.43, -0.21]
Academic identity → Digital Learning Perceived as Threat	-0.05	0.10	-0.54	.591	[-0.19, 0.19]
Social Belonging → Digital Learning Perceived as Threat	-0.25	0.10	-2.74	.006	[-0.45, -0.10]
System Justification → Maladaptive Coping with Failure	-0.10	0.10	-1.00	.317	[-0.28, 0.09]
Academic identity → Maladaptive Coping with Failure	-0.40	0.17	-2.34	.019	[-0.70, -0.05]
Social Belonging → Maladaptive Coping with Failure	-0.36	0.14	-2.61	.009	[-0.65, -0.11]

Note. Regression weights and 95% confidence intervals, based on 5,000 bootstrap samples. *b*-values indicate the unstandardized regression coefficients

3 Moderation analyses

Table 6

Regression analysis summary for system justification and first-Generation student status predicting maladaptive coping with failure

Variable	*b*	*SE*	95 % CI	β	*t*-value	*p*-value
Intercept	0.06	0.11	[-0.16, 0.28]		0.52	.608
System Justification	-0.32	0.09	[-0.51, -0.14]	-.19	-3.42	< .001
FGS-Status	-0.09	0.14	[-0.37, 0.19]	-.02	-0.63	.532
System Justification x FGS-Status	0.06	0.12	[-0.17, 0.30]	.03	0.53	.595

Note. $N = 848$, $F(3, 844) = 8.16$, $p < .001$, FGS-Status = First-generation student status, SE = standard error of *b*, CI = confidence interval.

Table 7

Regression analysis summary for academic identity and first-generation student status predicting digital learning perceived as challenge

**Variable**	***b***	***SE***	**95 % CI**	**β**	***t*-value**	***p*-value**
Intercept	0.01	0.07	[-0.12, 0.14]		0.15	.885
Academic Identity	0.23	0.09	[0.06, 0.40]	.15	2.71	.007
FGS-Status	-0.01	0.08	[-0.17, 0.16]	.00	-0.10	.919
Academic Identity x FGS-Status	0.22	0.11	[0.01, 0.44]	.11	2.02	.043

Note. $N = 848$, $F(3, 844) = 18.06$, $p < .001$, FGS-Status = First-generation student status, SE = standard error of *b*, CI = confidence interval.

Table 8

Regression analysis summary for academic identity and first-generation student status predicting maladaptive coping with failure

**Variable**	***b***	***SE***	**95 % CI**	**β**	***t*-value**	***p*-value**
Intercept	0.04	0.11	[-0.18, 0.25]		0.33	.742
Academic Identity	-0.34	0.15	[-0.63, -0.06]	-.13	-2.35	.019
FGS-Status	-0.07	0.14	[-0.35, 0.21]	-.02	-0.47	.638
Academic Identity x FGS-Status	-0.22	0.19	[-0.58, 0.15]	-.07	-1.17	.241

Note. $N = 848$, $F(3, 844) = 10.09$, $p < .001$, FGS-Status = First-generation student status, SE = standard error of *b*, CI = confidence interval.

Table 9

Regression analysis summary for social belonging and first-generation student status predicting digital learning perceived as threat

Variable	*b*	*SE*	95 % CI	β	*t*-value	*p*-value
Intercept	-0.04	0.06	[-0.16, 0.08]		-0.59	.554
Social Belonging	-.11	0.07	[-0.24, 0.02]	-0.09	-1.66	.097
FGS-Status	0.06	0.08	[-0.09, 0.21]	0.03	0.75	.453
Academic Identity x FGS-Status	-.21	0.09	[-0.38, -0.04]	-0.13	-2.44	.015

Note. $N = 848$, $F(3, 844) = 13.14$, $p < .001$, FGS-Status = First-generation student status, SE = standard error of *b*, CI = confidence interval.

Table 10

Regression analysis summary for social belonging and first-generation student status predicting maladaptive coping with failure

**Variable**	***b***	***SE***	**95 % CI**	**β**	***t*-value**	***p*-value**
Intercept	0.02	0.11	[-0.20, 0.24]		0.18	.859
Social Belonging	-0.09	0.12	[-0.33, 0.15]	-.04	-0.78	.438
FGS-Status	-0.03	0.14	[-0.31, 0.25]	-.01	-0.24	.814
Academic Identity x FGS-Status	-0.32	0.16	[-0.62, -0.01]	-.11	-2.03	.043

Note. N = 848, F(3,844) = 6.19, p <.001, FGS-Status = First-generation student status, SE = standard error of b, CI = confidence interval.

Table 11

Structural Equation Model: Alternative approach by starting with defensive strategies, their associations with threat reactions and threat of COVID-19

Regression	*b*	*SE*	*z*-value	*p*-value	95% CI
System Justification → Helplessness	-0.63	0.11	-6.25	< .001	[-0.81, -0.47]
Academic Identity → Helplessness	-0.24	0.31	-0.76	.450	[-0.56, 0.13]
Social Belonging → Helplessness	0.00	0.27	0.01	.992	[-0.34, 0.34]
System Justification → Digital Learning Perceived as Challenge	0.55	0.06	8.81	< .001	[0.44, 0.66]
Academic Identity → Digital Learning Perceived as Challenge	0.09	0.19	0.50	.621	[-0.11, 0.29]
Social Belonging → Digital Learning Perceived as Challenge	-0.05	0.17	-0.30	.762	[-0.26, 0.15]
System Justification → Digital Learning Perceived as Threat	-0.33	0.08	-4.25	< .001	[-0.43, -0.22]
Academic Identity → Digital Learning Perceived as Threat	0.04	0.29	0.14	.885	[-0.18, 0.27]

Social Belonging → Digital Learning Perceived as Threat	-0.40	0.25	-1.63	.103	[-0.61, -0.20]
System Justification → Maladaptive Coping with Failure	-0.23	0.12	-1.94	.053	[-0.42, -0.04]
Academic Identity → Maladaptive Coping with Failure	-0.23	0.37	-0.63	.530	[-0.62, 0.15]
Social Belonging → Maladaptive Coping with Failure	-0.37	0.31	-1.19	.236	[-0.71, -0.04]
Helplessness → Thread COVID-19	0.02	0.04	0.57	.569	[-0.06, 0.10]
Digital Learning Perceived as Challenge → Thread COVID-19	-0.20	0.06	-3.08	.002	[-0.32, -0.07]
Digital Learning Perceived as Threat → Thread COVID-19	0.66	0.08	8.91	< .001	[0.52, 0.81]
Maladaptive Coping with Failure → Thread COVID-19	-0.07	0.04	-1.84	.066	[-0.13, 0.01]

Note. N = 848, CFI = .874; RMSEA = .069; SRMR = .104. Regression weights and 95% confidence intervals, based on 5,000 bootstrap samples. *b*-values indicate the unstandardized regression coefficients
